# Response to Letter to the Editor: Japan Coma Scale and the Disorientation of the Nervous System

**DOI:** 10.2188/jea.JE20220266

**Published:** 2023-10-05

**Authors:** Mikio Nakajima, Yohei Okada, Tomohiro Sonoo, Tadahiro Goto

**Affiliations:** 1 Emergency Life-Saving Technique Academy of Tokyo, Foundation for Ambulance Service Development, Tokyo, Japan; 2 Department of Clinical Epidemiology and Health Economics, School of Public Health, The University of Tokyo, Tokyo, Japan; 3 TXP Medical Co. Ltd., Tokyo, Japan; 4 Department of Primary Care and Emergency Medicine, Graduate School of Medicine, Kyoto University, Kyoto, Japan

**Keywords:** Glasgow Coma Scale, Japan Coma Scale, clinical use, evaluation, conversion

**Response**

We thank Dr Saeki and colleagues for their interest and insightful comments pertaining to the clinical applications of the novel converting method we proposed.^1^ As correctly pointed out, the conversion table described in our study may not ensure absolute conversion of the Japan Coma Scale (JCS) to the Glasgow Coma Scale (GCS). As cautioned in the limitations, the proposed conversion method is not intended to serve as a blanket recommendation for use in the clinical setting. Instead, the primary purpose of this conversion table is to bridge the gap between Japanese and global epidemiological studies that are separated by a simple albeit obstructive difference in how deteriorations in consciousness are measured.

Saeki et al kindly pointed out that the conversion table defines both JCS 0 and JCS 1 as equivalent to GCS 15.^1^ We echo their cautionary message as the application of our proposed conversion table to clinical indications may inadvertently result in under-triage and potentially overlook patients with mild disorientation of consciousness. A single-point difference in the GCS (or JCS) could potentially incur critical consequences for an individual patient in the clinical setting; however, the proposed conversion table is designed for assessing risk stratification and identifying prognostic indicators at the population level. The GCS has been adopted in several severity classification scoring systems in the intensive care unit, such as the Acute Physiologic and Chronic Health Evaluation, the Sepsis-related Organ Failure Assessment, and the Simplified Acute Physiology Score.^2^^–^^4^ Using these scoring systems as precedence, a difference of a single point in GCS does not make a noticeable difference in the prediction of clinical outcomes.

With regard to the specific indictment that the intricate value of the JCS 1 not being granted adequate recognition in our conversion table, we respectfully submit that this is an inherent limitation of the GCS scoring system and not of the proposed conversion table. We concede that the JCS 1 score is beneficial in assessing subtle alterations in levels of consciousness and, furthermore, that a true equivalent to a score of 1 on the JCS does not exist on the GCS. One may surmise that the unique JCS 1 score classification culminates in fewer patients being overlooked in comparison with GCS-based assessments. However, the JCS and GCS are merely scoring systems designed to rapidly and universally assess consciousness levels. The categorical differences between the two systems do not infer a shortfall in the quality of the neurological assessments rendered by either JCS or GCS practitioners in the clinical setting.

Saeki et al rightfully address the limitations of our novel conversion method when applied to clinical use for evaluating individual patients. In contrast and by nature of design, the proposed conversion method was established with the humble hope that it could be broadly utilized by researchers conducting clinical and epidemiological studies to bridge the gap between JCS- and GCS-based investigations.
